# Recent advances and applications of nanostructured membranes in water purification

**DOI:** 10.55730/1300-0527.3635

**Published:** 2023-12-11

**Authors:** Didem AYDIN, İlkay Hilal GÜBBÜK, Mustafa ERSÖZ

**Affiliations:** Department of Chemistry, Faculty of Science, Selcuk University, Konya, Turkiye

**Keywords:** Pollution control, nanostructured membrane, nanofiltration, water treatment

## Abstract

In recent years, water pollution caused by hazardous materials such as metals, drugs, pesticides, and insecticides has become a very serious environmental and health problem that needs to be addressed urgently. The nutritional needs associated with the increasing population also increase the demand for water use and rapidly increase the rate of freshwater consumption. Since most of the water in the universe is in the form of sea water, which cannot be directly used, freshwater resources are limited, compared to the existing available water. When addressing the purification of all kinds of pollution in environmental research, nanostructured membranes attract attention as alternative solutions for water treatment. Nanostructured membranes, which can be used for filtration and water treatment process, are summarized in recent research. Various types of nanostructured membranes are presented and used to remove salts and metallic ions in water treatment processes. The representations and application areas of these membrane systems are explained. Consequently, new water treatment nanostructured membranes that can be developed and their effective separation performances are described. The benefits of nanostructured membranes for water treatment and their progress in purification are discussed.

## 1.Introduction

Water is the most important resource for the survival and development of all living things. The increase in the need for clean water, in parallel with the increasing human population, causes water scarcity and concerns more than 50% of the world’s population. Based on recent developments and changes, it has been emphasized that around 1 in 8 individuals lack the availability of secure drinking water [1]. As a result of rapid industrial development and increased human activity such as the use of fertilizers, pesticides and insecticides, mining, paper production, metalworking, various dangerous inorganic and organic pollutants are released into the environment, polluting the water and putting the ecological environment at risk. These organic pollutants lower the amount of dissolved oxygen in the water and endanger aquatic animals and ecosystems. Therefore, it is vital that immediate action must be taken without delay to address environmental pollutions, which threatens our world and our future [2].

Pollutants in water can reach the human body through the food chain and can cause long-term health risks. If we consider metal pollution, headaches in the central nervous system can be caused by mercury, and tissue damage can be caused by lead ions. Some metal ions can cause developmental delays, loss of appetite, and hearing disorders in children, especially during periods of growth and development [3,4].

In order to protect the environment, studies on successful water treatment systems and wastewater treatment continue today. Among the many separation procedures, more emphasis has been placed on membrane filtration due to its room temperature operation, simple operating procedures, low energy consumption, and high efficiency [5]. Membranes are classified according to their pore size, and nanostructured membranes perform very well in terms of water filtration. Most contaminants in wastewater (in the 1 to 10 nm range) can be filtered out, including metallic ions, salts, organic compounds, and microbes [6].

In this perspective, ultrafiltration and nano filtration of water and wastewater treatment using nanostructured membranes are the main treatment methods discussed in this review. In this review, nanostructured membrane types and synthesis strategies and the use of the membrane process for water and wastewater treatment are discussed. Also, nanostructured membrane application for removing various contaminants from water is discussed [7].

### 1.1. Membrane technology

Before membrane science and technology achieved its first significant industrial applications in the 1960s, it underwent a lengthy historical development through laboratory studies. Although the industrial application of synthetic membranes began in the 1960s, the study of the phenomenon of membranes can be traced back to the mid-18th century. The history of membrane science and technology has evolved through extensive laboratory experimentation and theoretical developments over the centuries, leading to significant breakthroughs in synthetic membrane technology. Since then, membrane-based processes have gained widespread acceptance and are increasingly being used in various industrial applications. However, with almost five decades of rapid advancement, membrane-based processes are now widely utilized in various industries and have proven to be highly beneficial for enhancing human life [7]. Loeb and Sourirajan’s 1962 production of high-performance, anisotropic, reverse osmosis (RO) membranes using asymmetric cellulose acetate (CA) represented a significant milestone in the industrial application of membrane technology. These error-free membranes boasted high flow rates and superior separation properties, making them highly attractive for a range of separation processes. This breakthrough played a pivotal role in the establishment of membrane technology as a critical separation tool in industry, paving the way for the development of increasingly advanced membrane materials and processes [8]. Subsequently, in the 1970s and 1980s, membrane technology experienced a significant expansion, and numerous scholars and experts held the belief that these advancements had the potential to resolve all issues pertaining to separation and reaction processes. Nanotechnology is considered a highly promising solution for addressing most challenges related to water treatment processes. Over the past few decades, the pressing demand for innovative membranes composed of precisely engineered nanomaterials has revolutionized conventional membrane concepts, leading to groundbreaking methods for water treatment [9]. Recently, with the advancement of nanotechnology and the development of new materials, more advanced membranes can be produced. These membranes can offer higher performance, higher durability, and higher selectivity properties [10]. Compared to other water treatment technologies like distillation, electrolysis, adsorption, and photodegradation, membrane technology emerges as the most superior. This is primarily due to its lower energy requirement for operation, high efficiency in separating substances, and ability to operate continuously. Numerous research studies have been carried out to enhance the overall performance of membranes, and nanotechnology has proven that by incorporating nanomaterials, the water permeability of membranes increases, their mechanical strength improves, and the occurrence of fouling is reduced [11].

### 1.2. Membrane processes

Membrane technology is widely used in various fields, and the membranes used in these applications have diverse structures, including solid dense or porous polymers, ceramic or metal films, liquid films/membranes with selective carriers or functionalized/charged barriers. High selectivity and fluxes in the membranes, good mechanical strength, chemical and thermal stability, low fouling tendency, and good compatibility with the working environment are the criteria that determine the performance of the membranes. Membrane production needs to be cost-effective and defect-free to ensure efficient performance [12]. Membranes are barriers that can be permeable or semipermeable, which means they permit certain substances to flow through while preventing others. In water treatment, the separation of contaminants relies on properties such as size and charge, which determine whether they can pass through the membrane [13].

Processes involving the use of membranes can be categorized according to the driving forces used during the process. Membrane separation processes used in various industrial applications can be classified as microfiltration, ultrafiltration, nanofiltration, and reverse osmosis. The categorization of water and wastewater treatment membranes is dependent on their rating of pore size and the pressure applied during the filtration process [14]. Microfiltration (MF) and ultrafiltration (UF) are types of low-pressure membrane systems that are operated within a range of between 10 and 30 psi. In contrast, high-pressure membrane process, which are nanofiltration (NF) and reverse osmosis (RO), require much higher operating pressures, ranging from 75 to 250 psi. Reverse osmosis uses a compact membrane with pores smaller than 1 nm [13,15].

Microfiltration (MF) membranes have a symmetric or asymmetric porous structure and separate particles and dissolved components based on a size exclusion principle. The transport of permeating across MF membranes occurs through convective flow through pores, which is a pore-flow/sieving mechanism. MF membranes, which have the largest pore size among practical membranes used in water purification (ranging from 50 to 500 nm), are typically used for pretreatment to enhance the efficiency of nanofiltration and reverse osmosis processes [16] . MF membranes are used to separate impurities with a size range of 0.1 to 10 μm from a solvent or other low molecular weight components, based on a sieving effect. Although some charge or adsorptive separation is possible, particles are mainly separated according to their dimensions. Compared to other filtration processes, the applied pressure in MF is relatively low, typically below 2 bars [17].

Ultrafiltration (UF) is a separation process that removes solutes larger than the solvent molecule by applying hydraulic pressure, forcing only the solvent to flow through a membrane with pore size in the range of 2 to 50 nm [14]. Permeation is inversely proportional to membrane thickness and directly proportional to pore size and applied pressure. UF membranes segregate macromolecules, colloids, and solutes with a molecular weight exceeding 10,000 from smaller molecules. Selectivity is governed by differences in size, surface charge, membrane properties, and hydrodynamic conditions.

Nanofiltration is a membrane separation technique commonly used in water purification. The membrane used in nanofiltration has a pore size of approximately 2 nm or less. Unlike reverse osmosis (RO) membranes, which have a nonporous structure and rely on a solution-diffusion transport mechanism, nanofiltration membranes operate at the interface of porous and nonporous membranes, using both sieving and diffusion transport mechanisms. Nanofiltration is considered a transitional separation procedure that falls between ultrafiltration and reverse osmosis [18]. The operating pressure of the NF membrane is typically in between that of the UF and RO processes. Nanofiltration has been found to be a highly efficient method of filtration, but in order to improve its effectiveness, it is often preceded by a pretreatment process using microfiltration or ultrafiltration [16].

**Figure 1:**
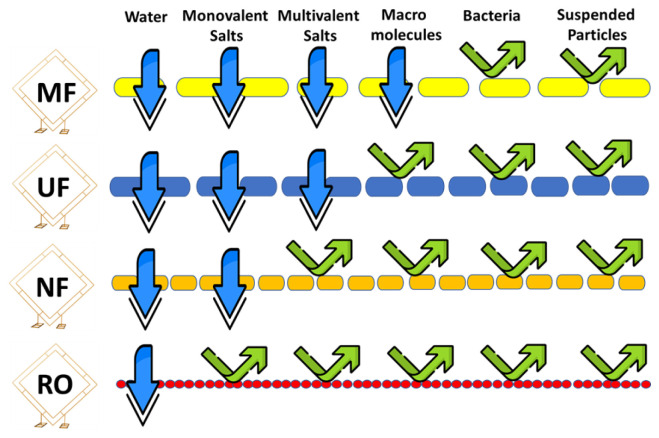
The permeability of different membrane filtration types.

Reverse osmosis is used for the separation of water from a solution by applying pressure exceeding the osmotic pressure. This results in the flow of pure water from the high solute concentration side through a membrane to the low solute concentration side. This process is termed “reverse osmosis” because it is the reverse of the normal osmosis process. This method is effective for removing total dissolved solids concentrations up to 20,000 mg/L. Reverse osmosis membranes, characterized by smaller pore sizes between 0.3 and 0.6 nm, are used in a process similar to nanofiltration. Like microfiltration, ultrafiltration, and nanofiltration, reverse osmosis is also a pressure-driven process. Reverse osmosis involves applying pressure to saline water as it passes through a semipermeable membrane, which allows water molecules to pass while retaining larger molecules like salt in higher concentrations [19]. The mentioned membrane filtration types are summarized in Figure 1.

## 2. Nanostructured materials for membrane development

Recent advancements in materials science and engineering have led to the development of novel membrane materials with improved performance and selectivity. Nanomaterials refer to a category of single materials that exhibit dimensions between 1 and 100 nm, comprising nanoparticles, nanofibers, and two-dimensional layer materials, among others. These materials display exceptional stability and possess a vast surface area, leading to outstanding permeation properties. Moreover, nanomaterials offer additional benefits that could enhance water purification processes. The use of advanced membranes based on nanomaterials is of significant interest due to their potential to address water scarcity and pollution issues, which are of growing concern worldwide. For example, nanostructured materials, which can be the most important ones, include carbon nanotubes (CNT), graphene oxide (GO), metal-organic frameworks (MOFs) etc. Ceramic membranes with nanostructured materials have shown great potential in improving the efficiency and selectivity of membrane-based technologies [13]. The integration of nanomaterials into membranes can be achieved through two main strategies: surface deposition and matrix embedding. These strategies enhance the physicochemical properties of the membranes and increase their efficiency. Such approaches enable the functionalization of membrane surfaces with specific properties, such as improved selectivity, durability, and permeability, addressing various challenges in separation processes. Moreover, the incorporation of nanomaterials into membranes has opened up new opportunities to develop high-performance membranes for various applications, such as water treatment, food processing, and energy production [20]. Despite the structure and configuration, membranes made from nanocomposite materials have a unique potential to contribute to the advancement of membrane development studies. The choice of dispersed and continuous phases is critical in creating nanopores for membrane development.

One of the key advantages of incorporating nanomaterials is their hydrophilicity, which can increase the water flux of membranes, leading to improved water recovery and reduced energy consumption. Additionally, nanomaterials possess high thermal stability, making them ideal for use in harsh treatment conditions and in a variety of wastewater treatment applications. Moreover, the surface roughness of nanomaterials can enhance the adsorption capacity of membranes, thereby improving the removal efficiency of contaminants such as heavy metals and organic pollutants. Nanomaterials also exhibit hydraulic stability, ensuring the durability of the membrane system over time. Another significant advantage of nanomaterials is their higher permeability, facilitating the transport of water and solutes through the membrane. They also offer effective fouling control, preventing the buildup of contaminants on the membrane surface and reducing the frequency of cleaning and maintenance. Finally, nanomaterials exhibit higher selectivity, enabling them to selectively remove contaminants from wastewater with minimal interference from other compounds present in the solution. Overall, the incorporation of nanomaterials into existing membrane technologies represents a promising strategy for improving the efficiency and effectiveness of wastewater treatment processes, with potential benefits for both the environment and human health [21,22].

In the separation process, the initial step involves choosing the material that possesses the desired properties. Subsequently, membranes specifically designed to target and separate environmental pollutants can be developed. It is crucial to possess a fundamental understanding of the materials and their utilization in constructing nanocomposite membranes. Characterization methods offer valuable insights into the latest advancements in nanomaterials and establish a relationship between their function and structure. Presently, cutting-edge membrane technologies make use of nanomaterials for water treatment. Among these, nanocomposite membranes stand out as a distinct category of membranes, characterized by a polymeric film surface that is either modified by or composed of nanoparticles. These membranes possess unique capabilities for effectively treating water in a dynamic manner. By harnessing the advantages of both the medium and nanoparticles, composite materials possess exceptional abilities [23,24]. A wide range of nanomaterials is utilized to prepare nanocomposite membranes, encompassing diverse forms and dimensionalities. The nanostructures most often utilized in the development or current employment of membranes for achieving high performance for water treatment are addressed. The properties of nanomaterial-based membranes are comparatively summarized in Table.

### 2.1. Metal-based nanoparticles

Metal-based nanoparticles have attracted significant attention in recent years for their potential applications in membrane technology. Metal nanoparticles, such as silver (Ag), gold (Au), platinum (Pt), and copper (Cu), have unique physicochemical properties that make them suitable for various membrane-related applications [25]. Nanoparticles (NPs) are increasingly being used in various industries such as electronics, material science, and energy production due to their unique properties. Among NPs, metal/metal-oxide NPs have gained significant attention from engineers and scientists for the production of nano-based products. The array of metal-oxide NPs includes both individual and binary oxides that have widespread industrial uses [26]. A variety of metallic nanoparticles (NPs) are commonly used in nanotechnology, including those made of precious and nonprecious metals. Among the most frequently used metal-oxide NPs are those composed of oxides of common metals, such as titanium, copper, zinc, iron, silicon, aluminum, cerium, nickel, magnesium, zirconium, indium, and lanthanum. These metal-oxide NPs exhibit unique properties, such as high surface area and reactivity, and are widely used in various applications, including catalysis, energy storage, environmental remediation, and biomedicine. Understanding the properties and potential toxicity of these NPs is crucial for their safe and effective use in different fields. Currently, metallic NPs can be synthesized and altered to possess a vast range of desired shapes and sizes using different chemical, physical, and biological methods [27,28]. Metal nanoparticles can also enhance the selectivity and permeability of membranes. For instance, the incorporation of copper nanoparticles into polymeric membranes has been shown to increase the separation efficiency of heavy metal ions. Similarly, platinum nanoparticles have been used to modify the surface of membranes to enhance their gas separation properties [29].

Polysulfone composite membrane with green-synthesized biogenic silver nanoparticles (Ag-NPs), using the spin coating technique in order to provide the antimicrobial activity of the Ag-NPs/PS composite membrane, was reported for the first time and tested using a direct contact test against bacteria such as *K. pneumoniae, P. aeruginosa*, *E. coli*, *E. faecium*, and *S. aureus*. Overall, this work provides a practical, cost-effective, and efficient approach for synthesizing Ag-NPs and utilizing them as an antimicrobial agent on the PS composite membrane [30]. In another study conducted by Qiao et al., Fe-doped TiO_2_/PSF composite ultrafiltration (UF) membranes were prepared using the phase inversion method. A visible-light responsive photocatalyst was incorporated into the PSF membrane casting solution, which was synthesized via the hydrothermal method. The photophysical and photochemical properties of the photocatalyst were investigated using various microscopic and spectroscopic techniques. Bisphenol A (BPA) was used as a model contaminant to evaluate the photocatalytic performance of the composite membranes, and the effects of various parameters, including metal variety, metal proportion, hydrothermal reaction time, temperature, and catalyst ratio, on the BPA removal performance were analyzed. The study found that the Fe-TiO_2_ photocatalyst had a higher BPA degradation rate than TiO_2_. Additionally, the resulting composite membrane exhibited simultaneous visible-light photocatalysis and ultrafiltration, with a high BPA removal rate, enhanced mechanical capacity and self-cleaning ability [31]. In a study conducted in 2014, a membrane reinforced with modified ZnO nanoparticles was developed and evaluated. The membrane was produced by adding modified ZnO nanoparticles to cellulose triacetate (CTA) membranes, which were manufactured using diffusion-induced phase inversion in dichloromethane (DCM). The transport efficiency of the membranes was optimized by adjusting the pH, concentrations of carriers in the membrane, Rhodamine B, and stripping phase concentrations. The results demonstrated that under optimized conditions, the transport efficiency reached 98% at pH 12. The incorporation of modified nanoparticles significantly enhanced the membrane’s features, as evidenced by the improved performance in transport efficiency. This study provides insights into the potential of modified ZnO nanoparticles as a reinforcement material for membrane fabrication [32]. In 2018, Kuvera et al. developed multifunctional membranes capable of performing both photocatalysis and filtration simultaneously. They embedded TiO_2_ nanoparticles Co-doped with N and Pd at different ratios into a PSf membrane using a simple phase inversion method. The resulting hybrid membranes exhibited enhanced porosity, wettability, and visible light activity while maintaining membrane integrity. However, higher TiO_2_ loadings resulted in increased membrane roughness and particle clustering. The hybrid membrane with 7% N,Pd TiO_2_/PSf showed up to 92% dye degradation under visible light irradiation at 180 °C. These solar-active photocatalytic membranes show promise for low-energy water purification in marginalized rural communities [33]. In 2021, Zhang et al. modified a traditional hydrothermal method to synthesize ultralong sodium titanate nanobelts, which were then converted to hydrogen titanate nanobelts through ion exchange. The researchers prepared self-standing flexible large-area membranes using these nanobelts and annealed them at different temperatures to obtain a self-standing TiO_2_ nanobelt membrane. Additionally, they prepared Cu-doped TiO_2_ membranes using ion exchange and postannealing. The pure sample exhibited visible-light responding photocatalytic activity due to self-sensitization of the methylene blue molecule, while UV-induced photocatalytic activity was higher due to photoinduced holes and electrons. Cu doping induced intragap states that decreased both visible and UV-induced photocatalysis [34]. Polyacrylonitrile-based activated carbon nanofiber composites with Fe_2_O_3_ and Co_3_O_4_ nanoparticles were reported by Kurukavak et al. (2021), and the composites showed high surface areas and were utilized for CO_2_ and CH_4_ adsorption with ACNF/Co_3_O_4_ having the highest adsorption capacity for both gases. The study suggests that ACNF-metal oxide composites are useful for designing adsorption systems for CO_2_ and CH_4_ [35]. In conclusion, metal-based nanocomposites have shown great potential in various fields, including catalysis, energy storage, and sensing applications. The incorporation of metal nanoparticles into the matrix of various materials has improved their physical and chemical properties, leading to enhanced performance. Ongoing research in this area is likely to lead to further advancements and expanded applications in the future [36].

### 2.2. Carbon-based nanoparticles

Carbon-based nanoparticles, also known as C-NPs, are widely used in various industries due to their remarkable properties, such as high thermal and electrical conductivity, as well as exceptional tensile strength. They are extensively employed in fields such as microelectronics, solar cell manufacturing, energy storage, and coating applications, among others. Thanks to their unique characteristics, C-NPs have become a vital component in many technological advancements, making them an essential material for modern industry. The exceptional characteristics of carbon-based nanoparticles, including their large surface area, adaptability for chemical and physical modification, ability to fine-tune properties for specific applications, and impressive capacity for removing both organic and inorganic pollutants, make them highly attractive for various water treatment applications. One of the key advantages of carbon-based nanomaterials is their large surface area, which provides ample sites for adsorption of various contaminants. Moreover, the surface of these materials can be easily modified through chemical or physical methods to enhance their adsorption properties and selectivity towards specific pollutants. This tunability has enabled the development of advanced carbon-based nanocomposites with enhanced removal efficiency for various contaminants, including heavy metals, organic compounds, and pathogens. Another notable advantage of carbon-based nanomaterials is their excellent antimicrobial properties, which have been attributed to their ability to generate reactive oxygen species upon exposure to light. These properties has been leveraged to develop photocatalytic materials that can effectively disinfect water, thereby mitigating the risk of waterborne diseases [37,38].

Carbon-based nanoparticles, such as carbon nanotubes, graphene, and fullerenes, have emerged as promising materials for addressing this challenge due to their unique physicochemical properties [39]. Carbon nanotubes (CNTs) are hollow, cylindrical structures made up of carbon atoms and are exceptionally strong and stiff. Graphene and graphene oxide are two-dimensional materials consisting of a single layer of carbon atoms arranged in a hexagonal lattice. Fullerenes are spherical carbon-based nanoparticles that have unique photophysical and photochemical properties, while carbon quantum dots are small carbon-based nanoparticles that exhibit unique optical properties. Carbon-based nanomaterials can be used for a variety of water treatment applications due to their unique properties, including their high surface area, ease of modification, and excellent capacity for removing both organic and inorganic contaminants. Researchers are continuing to explore new and innovative ways to utilize these materials to create sustainable and effective solutions for water treatment [40]. Some examples of carbon-based nanoparticles are shown in Figure 2a.

#### 2.2.1. Carbon nanotubes (CNT)

Carbon nanotubes (CNTs) are allotropes of carbon with cylindrical nanostructures, and they have unique electronic, mechanical, and thermal properties. CNTs can be single-walled (SWCNTs) or multiwalled (MWCNTs), and their properties are dependent on their diameter, chirality, and number of walls [41]. The surface chemistry of CNTs can be modified by functionalizing them with different chemical groups, such as carboxyl, hydroxyl, or amine groups, which can alter their solubility and reactivity. CNTs are widely used in various applications, including nanoelectronics, sensors, energy storage devices, and drug delivery systems. Additionally, CNTs represent another group of nanomaterials with great potential for membrane fabrication. They possess advantageous characteristics that make them highly desirable for various uses, including structural purposes, electronics, and energy storage. This is attributed to their exceptional performance in thermal and electrical conduction, mechanical strength, and impressive electronic features [42–44]. Studies using molecular dynamics simulations and theoretical models have demonstrated that water molecules in hydrophobic CNT inner channels are confined in a single-file manner, similar to the water wires observed in aquaporins [45,46]. Experimental observations have also shown that aligned CNT membranes, with their pore channels constructed by the inner walls, allow for ultrafast but selective mass transport, which is facilitated by the atomically smooth graphitic inner surface. On the other hand, nonwoven CNT membranes constructed by randomly stacking CNTs possess high porosity and high permeance, making them useful as a platform for other functional membranes, such as antibacterial and photocatalysis membranes [47–49]. CNTs exhibit a high aspect ratio, exceptional mechanical strength, low density, and high stiffness. Additionally, they undergo strong π stacking interactions with aromatic groups, leading to irreversible adsorption between hydrophilic units and CNTs. These attributes make CNTs highly suitable for utilization in low-pressure reverse osmosis (RO) membranes [35,36]. By modifying the surfaces of CNTs, their hydrophilicity can be enhanced through the incorporation of macromolecules or functional/hydrophilic components. This modification process facilitates the effective dispersion of nanoparticles and hydrophilic units, thereby improving the polar properties of the CNT surface. Consequently, the presence of hydrophilic groups establishes favorable bonding between CNTs and organic solvents that possess polarity. Moreover, the hydrophilic groups contribute to increased porosity with larger pore sizes and reduced frictional surfaces within the membrane structure. As a result, enhanced water permeability and exceptional pollutant rejection capabilities are achieved. Functionalization of CNTs using acids such as nitric acid, sulfuric acid, citric acid, polyacrylic acid, among others, introduces carboxylic groups onto the CNT surface. Employing alternative options such as polycaprolactone and polyvinyl pyrrolidone for acid processing further enhances the separation efficiency.

Recent advancements in fabrication techniques have allowed scientists to produce carbon nanotubes (CNTs) with remarkable precision, achieving dimensions as small as 1.6 nm in diameter and a length of several centimeters [42]. These enhanced manufacturing methods have resulted in CNT membranes that exhibit high water flux [50], making them an attractive material for reverse osmosis (RO) applications [51]. Molecular dynamics (MD) modeling has demonstrated that water flow through CNTs can be significantly greater than that predicted by continuum hydrodynamics [46,48]. Corresponding experimental investigations [50,51] have yielded similar findings. Numerous researchers have employed MD simulations to gain a deeper understanding of the mechanisms underlying ultrafast water movement. Kolesnikov et al. were the first to observe that water molecules confined within CNTs form an ice shell along with a chain-like water structure, indicating that water-molecule interactions play a more substantial role than interactions between water molecules and the CNT wall [52]. Subsequently, Joseph and Aluru explored the transport of water molecules across CNTs with varying degrees of hydrophobicity and surface roughness [48]. Their studies revealed that both hydrophobicity and the atomic smoothness of CNT walls are crucial for facilitating rapid water flow, enabling nearly frictionless movement. More recently, Falk et al. discovered that the coefficient of friction between the CNT wall and water molecules is closely linked to the curvature of the graphitic surface of CNTs [53]. Through MD simulations, it was demonstrated that the curvature influences the interaction energy landscape of water molecules on the CNT, with friction and CNT diameter exhibiting a direct proportionality that diminishes below a CNT diameter of 0.5 nm. Surprisingly, these studies indicated that water molecules flowing over the outer surface of CNTs experience higher friction.

Holt et al. reported an analysis on the pressure-driven flow of water through multiwalled carbon nanotubes (CNTs) with a diameter of 1.6 nm and observed the achievement of high water flux over the CNT membranes [51]. However, due to the relatively large diameters of the CNTs, they were unable to effectively function as molecular sieves for the rejection of salt ions. In a study by Shen et al. [54], thin film nanocomposite membranes were prepared using interfacial polymerization, and the membranes incorporated polymethylmethacrylate (PMMA) along with hydrophobically modified multiwalled carbon nanotubes (MWCNTs), which were fabricated in the presence of acid-modified MWCNTs (c-MWCNTs). The inclusion of PMMA/MWCNTs in the membranes led to enhancements in both selectivity and permeability. Multiwall carbon nanotube (MWCNT)/aromatic polyamide (PA) nanocomposite membranes were synthesized using a polymer grafting process by Shawk et al. in 2017. The resulting membranes were characterized, and it was shown that the addition of MWCNTs improved membrane strength, with monotonic increases observed in Young’s modulus, toughness, and tensile strength. Furthermore, the rejection of both salt and organic matter was improved with the incorporation of MWCNTs compared to the PA membrane without MWCNTs. In another reported work, a nanocomposite membrane fabricated by incorporating of 15 mg/g MWCNT in a 10% PA casting solution. The membrane rejected both NaCl and humic acid by factors of 3.17 and 1.67, respectively, compared to the PA membrane without MWCNTs. However, the addition of MWCNTs led to a decrease in membrane permeability by 6.5% [55].

Despite the significance of industrial-scale production of nanocomposite membranes for water treatment, it is crucial to evaluate their potential toxicity resulting from the exposure to integrated nanomaterials during the filtration process in order to mitigate adverse effects [56]. The toxic characteristics of nanoparticles embedded within a polymeric matrix can be attributed to factors such as their size, shape, charge, and production methods [57]. As previously discussed, both types of nanofillers, including carbon nanotubes (CNTs), exhibit potent antibacterial properties and exert toxicity through the release of ions and reactive oxygen species (ROSs), effectively eliminating bacteria via oxidative stress stimulation [58]. This exceptional attribute enables the application of CNTs in composite ultrafiltration (UF) membranes for various purposes, enhancing the efficacy of filtration processes [59]. Numerous studies have demonstrated that CNTs contribute to improved water filtration by effectively removing nonpolar and micro- and macrosized contaminants, salts, as well as chemical waste materials [60].

In conclusion, the utilization of carbon nanotubes has demonstrated significant potential in diverse fields. Their remarkable physical and chemical characteristics render them flexible materials for deployment in composites, sensors, electronics, and membrane technologies. Continuous research and innovation in the realm of carbon nanotubes are expected to bring forth more improvements and broader applications in the times ahead [61].

#### 2.2.2. Graphene oxide (GO)

Graphene is a carbon-based material with a hexagonally structured lattice of sp2 bonded atoms, which has promising properties for membrane technologies. It can be used to fabricate high-flux membranes with size-adjustable pores for molecular sieving purposes, and large-scale production is possible [62,63]. The exceptional electronic characteristics of graphene have led to its increased popularity. In its pristine state, graphene exhibits superior breaking strength and is impermeable to molecules as small as helium. Therefore, it can be used to fabricate ultrathin high-flux membranes with size-adjustable pores for molecular sieve purposes. Large-scale membrane manufacturing is also possible due to the production of single- or multilayered sheets of graphene over large areas, including 30-in sheets maintained on a roll-to-roll principle. Several studies have explored the factors causing defects in the electrical characteristics of graphene. Recent simulations and experiments have demonstrated that the pores in subnanometer sizes can be controlled via techniques such as oxidation, electron beam irradiation, ion bombardment, or doping [64].

Graphene oxide (GO) is a single atomic layer material composed of carbon, hydrogen, and oxygen molecules that can be easily produced through the oxidation of readily available graphite crystals. It is water-soluble and easy to process, making it a promising material for use in composite applications. The unique properties of GO include its low cost, high efficiency of production using inexpensive chemical methods, and its ability to form highly hydrophilic colloidal suspensions. Graphene layers consist of sp2-bonded carbon atoms arranged in a perfectly flat manner with microscopic fluctuations. The oxidation of graphene with strong oxidizing agents introduces oxygen-containing functional groups into the structure, which not only expands the layer separation but also renders the material hydrophilic, allowing it to disperse in water. Sonication of graphite oxide in water leads to exfoliation, resulting in the production of few-layered and single-layered graphene, also known as GO. Graphene oxide has gained attention as a promising material for membrane applications due to its unique properties. GO has a layered structure similar to graphite, but with abundant oxygen-containing functional groups that make it hydrophilic and easily dispersible in water [65]. This property allows GO to be easily processed into thin films or coatings on various substrates for membrane fabrication. The high surface area and large number of functional groups in GO also make it an excellent candidate for selective molecular separation. Graphene has emerged as a promising material for water remediation due to its high fluid permeability and size-selective transport properties. It offers greater efficiency and cost-effectiveness compared to standard nanofiltration membranes. Pristine graphene, graphene oxide, and reduced graphene oxide can be used for water desalination, and graphene membranes can be manufactured in single, stacked, and multilayer forms [66]. Monolayer graphene coated on a porous substrate has been experimentally and theoretically investigated for nanofiltration, with intrinsic pores ranging in size from 1–15 nm, allowing for size-selective passage of molecules.

Several studies have demonstrated the potential of GO membranes for various separation applications, including water desalination, gas separation, and solvent nanofiltration. For example, Peng et al. [67] developed GO membranes for CO_2_ separation and achieved high separation performance due to the selective transport of CO_2_ through the nanochannels in the GO sheets. Akin et al. developed a simple and eco-friendly enzymatic-reaction-based method to produce a reduced graphene oxide/polyaniline composite material. This material was incorporated into polysulfone membranes using phase inversion polymerization. The incorporation of rGO resulted in a hydrophobic membrane surface with enhanced macrovoids, while the rGO/PANI-incorporated membrane surface exhibited a partly hydrophilic nature due to the presence of PANI fibers. The membranes exhibited improved salt rejection after rGO/PANI doping, reaching a maximum of 82% NaCl rejection at 10 bar applied pressure. The PSf-rGO/PANI composite membrane showed the highest mean porosity and water flux [68]. In their study, Sint et al. [69] used molecular dynamics (MD) simulations to investigate the movement of ions through graphene membranes with pores of approximately 0.5 nm, which were terminated with nitrogen and hydrogen. They found that the nitrogen-terminated pores allowed for the passage of Li^+^, Na^+^, and K^+^ ions, while the hydrogen-terminated pores allowed for CI^−^ and Br^−^ ions, but not F^−^. Interestingly, they observed that although smaller ions were able to transport more easily, they had lower passage rates due to their strongly bound hydration shells. The flux over the graphene pores was found to be twice that of carbon nanotubes (CNTs), but with a higher resistance to transport due to the entry locations of the pores. The researchers also noted that the potential for graphene membranes in water desalination is much greater than that of polymeric reverse osmosis (RO) membranes, with water fluxes up to ten times higher. According to various studies [52,70,71] the presence of functional groups, including hydroxyl (−OH), carboxyl (−COOH), and epoxy (C-O-C) on graphene oxide (GO) nanoparticles makes their distribution more favorable than basic graphene. These functional groups promote the fabrication and utilization of GO nanoparticles in membranes. Experiments have shown that GO exhibits high water flux in membrane fabrication, possesses antifouling characteristics, and demonstrates good resistance against chemical degradation in reverse osmosis (RO) and nanofiltration (NF) membranes. The polar oxygen-containing functional groups on GO provide hydrophilic properties, enabling it to exfoliate in some solvents and disperse in water under sonication. However, to enhance its incorporation within the polymer matrix, functionalization of GO is necessary. Flexible polymers with functional groups can be utilized to achieve well-defined nanostructures for GO assembly. Furthermore, the incorporation of GO in high-strength membranes enables the creation of reliable modules with improved stability in practical applications.

GO nanocomposites, when combined with other nanomaterials and polymers, have shown promise in enhancing the efficiency of desalination processes. The addition of GO sheets using an additive method allows for the creation of membranes with varying sizes and high-flux in both RO and filtration membranes. However, the exclusion of salt in these membranes can range from 6% to 46% due to the occurrence of unzipped slits during the membrane fabrication process [52]. Recently, Fathizadeh et al. [72] demonstrated a new approach to membrane fabrication by implementing ultrathin membranes with GO through inkjet printing onto a modified polyacrylonitrile (M-PAN) support. This membrane exhibited improved pure water flux and rejection rates compared to commercially available NF membranes. Another perspective for enhancing the hydrophilic characteristics of GO involves functionalization. This alternative method for membrane fabrication has shown promising results.

Despite the numerous advantages of monolayer graphene membranes in nanofiltration for water desalination, the commercial-scale production of nanoporous graphene remains exceedingly complex, and single-layer graphene lacks the strength to withstand standard filtration pressures. Conversely, multilayered graphene oxide (GO) membranes can be manufactured at a commercial level and possess the necessary durability to endure the commonly applied pressures. The layered GO nanosheets feature nanochannels that are modified with diverse polar functional groups, facilitating water permeation within the membrane [73]. Taking advantage of the remarkable slip behavior of water within the interlayer channels, the stacked GO hinders the passage of solute particles. In terms of selectivity, the interlayer spacing distance of the GO nanosheets increases to 0.9 nm when immersed in an ionic solution, attributed to the hydration effect. This structural alteration allows for the permeation of K^+^ and Na^+^ ions while impeding the membrane for the purpose of desalination [74].

The development and optimization of GO membranes continue to pose significant challenges, despite their promising properties. A major hurdle in this regard is the precise control of pore size and distribution, as it has a direct impact on the separation efficacy of the membranes. To overcome this obstacle, several techniques have been suggested, such as chemical modification, physical confinement, and template-assisted synthesis, to tailor the pore size and distribution in GO membranes.

#### 2.2.3. Carbon quantum dots

Carbon quantum dots (CQDs) are a type of carbon-based nanomaterials that have unique properties due to their nanoscale size and surface functional groups. Nanoparticles known as carbon nanotubes generally have diameters ranging from 3 to 20 nm and feature semispherical shapes with either amorphous or nanocrystalline structures. Their initial discovery dates back to 2004, during the purification process of single-walled carbon nanotubes (SWCNTs) [75]. The presence of epoxy, hydroxyl, and carboxyl groups on their surfaces enhances their water solubility, which is an advantage for their functionalization and helps to passivate their surfaces. These functional groups also provide CQDs with antifouling ability, enhanced chemistry, significant chlorine resistance, and antimicrobial properties. Due to their hydrophilic groups, CQDs have been found to be effective in minimizing the usage of CQDs in membranes, while maintaining low cost and extraordinary separation efficiency. This is because the hydrophilic properties of the CQD nanomaterials can change the negative charges of the membrane, repel foulants, and reduce membrane roughness. When incorporated into polymeric membranes, both thin-film composite and mixed matrix membranes have shown increased hydrophilic properties and mechanical strength. Therefore, CQDs have emerged as promising candidates for the development of high-performance membranes in various applications, such as water treatment, gas separation, and energy production. The unique properties of CQDs and their ability to modify membrane surfaces make them a viable option for improving membrane performance, with potentially significant implications in addressing global challenges related to water scarcity and environmental pollution [76].

Carbon quantum dots (CQDs) possess favorable characteristics for the creation of multifunctional composite materials owing to their small size on the nanoscale, rich chemistry, and antifouling properties. Their unique sizes, shapes, and surface chemistry enable them to disperse effectively in polar solvents such as water, ethylene glycol, and N-methyl-2-pyrrolidone, as well as in polymer matrices. These characteristics are crucial for their application in membrane formation and separation. Consequently, the development of CQD-modified membranes has been explored to enhance membrane properties and improve performance in various ways [76]. Fathizadeh et al. [72] prepared thin film nanocomposite membranes to use in the RO process by interfacial polymerization using TMC and phenylenediamine, and implemented polysulfone ultrafiltration membrane as a support layer. They fabricated nitrogen-doped GO quantum dots (N-GOQD). The embodiment of 0.02 wt% N-GOQD in polyamide membranes tripled the water flux, and there was no loss in salt rejection compared to pristine polyamide membranes due to improved surface hydrophilicity and active sites. In a recent study by Yang et al., novel nanofiltration (NF) membranes were developed for the purification of amino acids from biogas slurry. These membranes incorporate hydrophilic carbon quantum dots (CQDs) as the interlayer, which react with polyethyleneimine (PEI) to create lower crosslinking degrees and fast water and ion transport channels in the interfacial polymerization layer. These membranes exhibited high selectivity, achieving rejection rates above 90% for model amino acids such as lysine, glutamic acid, and leucine, while allowing unwanted salt ions to permeate through. These results demonstrate the great potential for NF membranes in the valorization of biogas slurry [77]. In an effort to address the growing issue of global contamination of surface and groundwater by arsenic (As) and selenium (Se), researchers have developed thin-film nanocomposite (TFN) membranes incorporating sodium ion-modified carbon quantum dots (Na-CQDs). These ultrafine Na-CQDs were synthesized through the pyrolysis of citric acid and then incorporated into a polyamide (PA) layer during interfacial polymerization. The resulting Na-CQD modified TFN membranes exhibit higher surface hydrophilicity and reduced pore sizes, resulting in increased pure water permeability (PWP) and rejection rates for toxic ions. Moreover, these novel membranes demonstrate superior antifouling properties and long-term stability over a 180-h test period. This study provides valuable insights into the development of nanoparticle-modified polymeric membranes for water purification [78].

In addition to their unique characteristics, CQDs also have potential applications in other various fields such as bioimaging, biosensors, optoelectronics, drug delivery, and photocatalysis due to their size-dependent optical and electronic properties. They can be synthesized from various carbon sources, such as coal, biomass, and waste materials, using different methods, including hydrothermal, microwave, and chemical methods. CQDs are also biocompatible and can be used as fluorescent probes in bioimaging due to their low toxicity and high photoluminescence quantum yield [79]. In a study conducted by Baslak et al., they report the synthesis of a composite material consisting of polymer-coated quantum dots (QDs) and carbon nanotubes (CNTs) that exhibit high biocompatibility and low cellular toxicity. The results showed that the quantum dots were well dispersed on high-density nanotube surfaces. The toxicological evaluations of the QDs and MWCNT-QD-polymer hybrids were performed on human breast carcinoma cells, and fluorescent imaging of these materials in a live cell system was conducted. The MWCNT-QD-polymer hybrids exhibited a strong red fluorescent signal under confocal microscopy and good fluorescent stability for 6 h in the live cell system. The toxicity comparison of QDs and MWCNT-QD-polymer hybrids showed that the presence of a thin coating of PGMA on the MWCNT-QD hybrid surface reduced cellular toxicity and increased biocompatibility [80].

Researchers have been studying the development of CQD-based membranes for water treatment due to their high filtration efficiency, environmental friendliness, and easy synthesis process. CQD-based membranes have shown exceptional performance in removing impurities from water, making them a promising technology to address global water scarcity and pollution challenges. The sustainable and straightforward synthesis process of CQD-based membranes makes them a promising alternative to traditional membrane technologies. The continuous advancement of CQD-based membranes has opened up new opportunities for their use in energy storage, optoelectronics, and biomedical imaging. CQD-based membranes hold great potential for advancing the field of water treatment and ensuring access to safe and clean water worldwide [78].

### 2.3. Metal-organic framework (MOF)

Metal-organic frameworks (MOFs) are a type of nanoporous inorganic-organic hybrid materials formed by connecting metal ions or clusters with organic ligands. MOFs possess unique properties, such as high crystallinity, large specific surface area, high porosity, diverse and adjustable structure, and flexibility, making them highly attractive for a range of applications, including gas storage and separation, catalysis, sensing, drug delivery, and environmental remediation. The ability to precisely control their properties by selecting appropriate metal ions and organic ligands has led to the design and synthesis of MOFs with specific properties tailored for particular applications. The structural diversity of MOFs also allows for the fabrication of MOF-based composites with specific frame structures by rational monomer design and synthetic assembly. The organic ligands of MOFs are usually long with ρ-bonds, and the common coordination bonds are also similar to ρ-bonds, contributing to their flexibility and rich physical and chemical functions. Additionally, the flexibility of MOFs can lead to peculiar properties, such as particular physicochemical changes and multistep adsorption/desorption processes [5].

Membranes containing metal-organic frameworks (MOFs) have emerged as a promising approach for water treatment. These membranes are created by growing polycrystalline MOF layers onto polymer substrates, which provide strong mechanical support and enhance the performance of the membranes in various applications. MOF-containing membranes offer regular and adjustable pore sizes, excellent selectivity and permeability, as well as good compatibility with polymers, making them highly attractive for water remediation applications. These membranes were first prepared in 2005, and since then, they have been extensively studied and developed due to their potential value in practical water remediation [81]. Furthermore, the excellent compatibility between the MOF layers and the polymer substrates has contributed to the increased mechanical strength and stability of the resulting membranes, making them more robust and suitable for practical water treatment applications. These membranes have shown remarkable characteristics such as tunable porosity, high selectivity, and permeability, which enable them to efficiently filter and separate various contaminants from water. The regular and ordered structure of the MOF layers further enhances the membrane’s performance, making it more effective and reliable in water remediation processes. The MOF layer on the polymer substrate provides regular and tunable porosity, which enables high selectivity and permeability for specific molecules [82,83]. Figure 2b shows an example of a typical MOF structure, highlighting the metal nodes and organic linkers that comprise the framework.

Studies have shown that MOF-containing membranes have excellent performance in water treatment applications, such as desalination, removal of heavy metals and organic pollutants. Pan et al. developed a zeolitic imidazole framework (ZIF-8) membrane using a hydrothermal seeded growth method for propylene/propane separation. The membrane exhibited exceptional separation performance for a wide range of propylene/propane mixtures with a propylene permeability of up to 200 bar and a propylene to propane separation factor up to 50. The separation performance of the ZIF-8 membrane surpassed the upperbound trade-off lines of existing carbon and polymer membranes. Additionally, the membrane demonstrated excellent reproducibility, long-term stability, and thermal stability in experimental tests [84]. Liu et al. employed an in situ solvothermal synthesis method to prepare continuous Zr-MOF membranes. These membranes demonstrated remarkable rejection of multivalent ions (achieving up to 99.3% for Al^3+^), accompanied by moderate permeance and good permeability. Moreover, the exceptional chemical stability of UiO-66 material was evident from the preservation of membrane performance upon exposure to diverse saline solutions for up to 170 h. Considering the impressive separation performance and stability, the newly developed UiO-66 membrane shows great potential for water desalination applications [85]. Pal et al. synthesized an aluminum metal-organic framework (Al-MOF), [Al(OH)(BPDC)] (DUT-5), and characterized its high surface area and micropores using N_2_ gas sorption measurements. The thermal stability and phase purity of DUT-5 were also investigated. DUT-5 was successfully incorporated into chitosan (CS) polymer to prepare a mixed matrix membrane (MMM) for the pervaporation of water/ethanol. The DUT-5@CSDUT-5@CS membranes with 0.15wt% loading showed a significant increase in permeability and separation factor compared to the CS membrane. These promising results indicate the potential of using microporous Al-MOF in chitosan MMMs for bioethanol separation processes [86]. Fang et al. developed a zirconium-based metal-organic framework (MOF) membrane for wastewater separation. The membrane was constructed using a flexible substrate and UiO-66 MOF coating. It exhibited high rejection rates for various dyes and antibiotics, as well as promising antifouling properties and stability. The study suggests the potential of the UiO-66/PGP thin-film composite (TFC) membrane for industrial and pharmaceutical wastewater treatment [87]. In conclusion, the MOF-containing membranes have emerged as a promising technology for water treatment and purification, and continued research and development in this field holds great potential for future applications.

### 2.4. Functionalization of nanoparticles

Functionalization of nanoparticles refers to the process of modifying the surface of the nanoparticles with various functional groups or biomolecules to tailor their properties and improve their performance for specific applications. There are several methods for functionalizing nanoparticles, including chemical functionalization, physical adsorption, electrostatic interaction, and covalent bonding [88]. Chemical functionalization involves attaching functional groups covalently to the surface of nanoparticles. This approach yields a strong and stable bond, but controlling the orientation and density of the functional groups can be difficult. Physical adsorption is a simpler and more flexible method where functional molecules are attached noncovalently to the surface of nanoparticles. However, the molecules may detach over time due to the weak bond. Electrostatic interaction involves attaching charged biomolecules, such as DNA or proteins, to the surface of nanoparticles via interaction between oppositely charged particles. Covalent bonding forms a strong and stable chemical bond between functional molecules and nanoparticles, providing control over the orientation and density of functional groups. However, achieving uniform coverage of nanoparticles with functional groups can be challenging [88,89]. It is noteworthy that nanoparticles cannot be used directly on their own because they often exhibit certain harmful effects on the surrounding biological environment. To address this issue, the surface functionalization of nanoparticles has been extensively investigated and proven to play a critical role in developing nanoparticles for practical applications [90].

Selective surface modification allows the combination of nanoparticles that may or may not be successfully utilized in a system for analysis, detection, imaging, therapeutic, or diagnostic purposes. For these nanoparticle systems, various ligands can be added to the nanoparticles depending on which effect is desired, enabling the development of single-function or multimodal capabilities. For instance, bulky ligands and hydrophobic molecules can be added to nanomaterial surfaces to prevent nanoparticle core aggregation, while surfaces for use in aqueous environments can be coated with water-soluble polymers such as polyethylene glycol (PEG) to improve solubility and biocompatibility [90,91]. In a conducted study, PVDF membranes were modified using polyacrylic acid and titanium dioxide nanoparticles to enhance their antifouling properties. Two different methods were used to immobilize TiO_2_ on the membrane surface: self-assembly and grafting from acrylic acid monomers. The grafting from method showed better dispersion of TiO_2_ and higher grafting yield. The modified membranes showed improved resistance to fouling when tested with whey solution. The stability of the nanoparticles during operation and cleaning was confirmed due to their covalent attachment to the PAA network [92]. Adding nanoscale additives to PBI (polybenzimidazole) membranes is a promising method for improving proton conductivity and mechanical stability during acid doping and MEA (membrane electrode assembly) fabrication. However, the use of inorganic materials can reduce proton conductivity. To overcome this issue, researchers have used functionalized nanomaterials to form proton conducting pathways. For example, imidazole-functionalized silica particles have shown promise in improving acid retention and proton conductivity. Similarly, sulfonated silica nanoparticles and polyvinylimidazole/poly(sulfonated vinyl-benzene)-modified SiO have been effective in increasing proton conductivity while reducing methanol permeability [93,94]. In a study, an ozone-mediated approach was employed to synthesize PBI-functionalized silica nanoparticles (SNP-PBI), utilizing N-(p-carboxyphenyl)maleimide (pCPM) modified silica (SNP-pCPM) as starting materials. The presence of PBI chains in SNP-PBI was anticipated to enhance the compatibility between PBI and SNPs and to stimulate the formation of proton conducting channels within the membranes. Through the fabrication and characterization of PBI/SNP-PBI nanocomposite proton exchange membranes, it was found that the modified membranes displayed improvements in mechanical strength, proton conductivity, and single cell performance in comparison to the pristine PBI membrane [94,95].

Nanocomposite membranes exhibit greater efficiency in removing contaminants and higher water flow compared to the intact membrane. Functionalization is a critical approach for modifying the surface properties of nanoparticles in materials science and engineering. This technique allows for the introduction of functional groups, which can enhance the solubility, stability, and selectivity of nanoparticles. Additionally, functionalization can improve the biocompatibility and bioavailability of nanoparticles, thereby leading to increased usage in biological systems. The application of functionalized nanoparticles holds great promise for the development of advanced materials with customized properties and functions, enabling their application in a broad range of fields, including biomedicine, energy storage, and catalysis [96].

## 3. Nanomaterial-based polymeric membrane

### 3.1. Block copolymer (BCP)

Block copolymers are important in nanomaterial-based polymeric membranes as they provide unique structures created by combining different polymer blocks. These distinct block properties enable membrane customization for specific applications. When one block is hydrophobic (repelling water) and the other is hydrophilic (attracting water), it allows control over membrane surface and porosity. This enhances membrane performance in applications such as water purification and separation. Thus, block copolymers are crucial for developing nanomaterial-based polymeric membranes [97]. Block copolymers have a wide range of applications, including in nanotechnology, drug delivery, and coatings. [98]. They can also be used as templates for the synthesis of nanoparticles, which can have controlled size and shape. Additionally, BCP-generated pores can be inverted in polarity and size by incorporating smart and functional polymer segments [99,100]. The membrane preparation process involves the application of BCP annealing and subsequent UV etching steps, as well as the formation of the different types of BCP embedded vertically or horizontal on a membrane substrate. BCPs with immiscible blocks are separated into microphase domains controlled by the Flory-Huggins interaction parameter between the blocks and the polymerization degrees of each block that govern the equilibrium domain size (generally 3–100 nm) and morphology. BCPs can self-assemble into spheres, cylinders, gyroids, and lamellar structures. The microphase separation forms in BCPs, particularly cylinder or gyroid shapes, have attracted the attention of researchers working in the field of membranes, as they can create nanometer-sized high-density pores controlled by the chemistry and thermodynamics of the copolymer. To provide effective, high selective, high-yield, and mechanically resistant water purification, the membrane’s geometry and dimensions should be carefully designed, considering the application requirements [99]. Figure 3a shows the formation method of a block copolymer.

In block copolymers, coating refers to the formation of a thin layer of one block on the surface of the other block. The thickness of the coating layer can vary depending on the strength of the interaction between the block and the surface, as well as the molecular weight and concentration of the block copolymer [100]. Swelling in block copolymers refers to the ability of the polymer chains to absorb a solvent and increase in volume. This is due to the presence of one block that is more soluble in the solvent than the other block. The degree of swelling can be controlled by changing the solvent composition, temperature, and the composition and molecular weight of the block copolymer [101]. Both coating and swelling are important factors in the self-assembly and functionalization of block copolymers. For example, coating can be used to create surface patterns and modify surface properties, while swelling can be used to create pores and channels within the copolymer structure for various applications such as drug delivery and nanofiltration [102].

In 2014, Buzoglu et al. synthesized biocompatible PS-b-PGMA diblock copolymers with different molecular weights using ATRP. They investigated the effects of synthesis parameters on the copolymerization and used the copolymers to interact with fluorescent blood proteins, which were quantified through fluorescence spectroscopy. The PS-b-PGMA diblock copolymers were able to immobilize blood proteins, and the subsequent drug interactions were characterized using various analytical techniques. The results showed promise for the use of these copolymers in drug delivery systems, as they demonstrated biocompatibility and potential for targeted delivery of drugs to specific cells or tissues [103]. Figure 3b shows the schematic illustration of the synthesis of the PS-b-PGMA diblock copolymer.

Dmitrenko et al. developed high-performance membranes made of a block copolymer of PDMS and PPO for enhanced dehydration of ethanol. The membranes were modified with graphene oxide and demonstrated optimal transport characteristics. The synthesis was confirmed by FTIR and NMR, and changes to morphology and physicochemical properties were studied. This study is significant in the field of renewable energy as it presents a novel approach for the purification and concentration of bioalcohols, offering a promising alternative liquid biofuel. The results of this study show that the modified BCP membranes achieved optimal transport characteristics, such as high permeation flux, selectivity, and pervaporation separation index, making them highly efficient for the dehydration of ethanol over a wide concentration range [104]. A graphical summary of this study is presented in Figure 3c.

Luo et al. developed a novel membrane-based technology for oil/water separation and aqueous solution filtration, using a vertically oriented nano porous block copolymer membrane with a uniform pore size of approximately 23 nm and high nanopore density. The membrane exhibited a moderately high throughput rate and broader applications for molecular weight dependent filtration of water-soluble polymers [105]. The membranes, synthesized from PDMAEMA-b-PS BCPs, were immobilized with AuNPs and used to catalytically transform nitrophenol to aminophenol with a catalytic efficiency up to 100% [106].

## 4. Challenges and future perspectives

In order to achieve the realization and implementation of nanostructured membrane approaches, several significant milestones that must be attained. Nanomaterials play a crucial role in developing the next generation of membranes for water treatment and other applications systems. Nanoparticles, CNT, TiO_2_, Ag, GO, etc., and various 2D materials such as covalent triazine frameworks, MOFs exhibit subnanometer-sized pores suitable for water treatment and require optimization of their pore sizes to enhance their high-flux performances. Moreover, nanofibers and graphene nanosheets have been extensively studied over the past decade for this purpose to develop the membranes that have controlled nanopores. These materials possess adjustable pore sizes, exceptional porosity, and open porous structures, enabling energy-efficient and cost-effective water treatment processes. As a result, they have gained considerable attention for constructing state-of-the-art for membrane developments such as ultrafiltration, nanofiltration, and reverse osmosis membranes, which serve as permeable solid supports for the selective layer. Despite their merits, nanostructured membranes have not yet been implemented on an industrial scale. This limitation may stem from the lack of proper and reliable membrane development methodologies that incorporate nanomaterials which perform the contact nanostructures.

Despite the impressive progress, there are still many challenges that need to be addressed. The challenges are as follows:

- It is still difficult to achieve uniform distribution of nanomaterial within membranes on a large scale. However, by selecting one or more suitable combinations of functionalization processes, it would be possible to prepare large quantities of high-quality and robust membranes.
- There is still great potential for reducing the hydrophobicity of the membrane surface, especially for highly toxic aqueous dispersants.
- It is desirable to develop a reusable membrane that can target different kinds of effluent and survive for a very long time, thus avoiding replacement cost. Modification of membranes with Ag NPs, TiO_2_ NPs, etc., possessing intrinsic biocidal activity, can enhance antifouling performance of membranes, undoubtedly improving the sustainability and antimicrobial resistance. However, the toxicity of released metal ions toward aquatic animals remains a serious concern.
- Membrane development using nanomaterials, such as NPs, nanotubes, nanocomposites, and organic metal frameworks, still presents challenges that require more systematic and practical implementation. Achieving appropriate integration of these materials is crucial, and the nanomaterials employed for membrane modification must be effectively adhered to or embedded within the membrane.
- Fouling, a critical factor in membrane development, significantly inhibits the widespread utilization of membranes in large-scale applications. Therefore, it is imperative to appropriately tackle this problem in order to enhance the effective utilization of membranes in various applications.
- Despite significant advancements in the field, there are several areas of research that require further investigation. Regarding nanostructured membranes, while extensive theoretical and experimental studies have been conducted on ion sieving through graphene nanopores, exploration of other types of 2D materials is also needed to study. Additionally, attention should be given to understanding the nature of pore chemistry and improving the controlled pore fabrication process.

## 5. Conclusion

Industrial growth, population increase, and climate change have emphasized the need for efficient water resource management. While membrane technology serves as a highly effective water treatment method, challenges such as fouling, selectivity, and permeability hinder its widespread use. To overcome these limitations, the use of advanced materials is crucial for enhancing membrane performance [107].

The practical implementation of nanostructured membranes for various real-world conditions, including chemical, thermal, and mechanical challenges, requires long-term testing. Unfortunately, in laboratory settings, these membranes are often tested with a single type of pollutant, overlooking the real-world scenario of multiple pollutants, such as dyes, ions, or organics [108]. Graphene membranes, with their potential for exceptional water permeability and ionic selectivity comparable to conventional membranes like NF or RO, have mainly been explored in theoretical studies, with practical applications still in development. As we advance in developing cutting-edge water treatment processes using nanomaterials, it is crucial to approach their implementation with caution [109]. Nanostructured membranes show great promise in water purification, particularly for water treatment and desalination. Combining nanomaterials with commercial membranes enhances separation performance; however, standardization efforts are needed due to research variability. While ideal separation membranes are on the horizon, the journey to commercial use remains challenging. Balancing production costs with simplicity is a key challenge. In summary, nanomaterials offer a solution to the global water crisis.

This review highlights the use of different nanomaterials in membrane fabrication for water treatment. It underscores the significance of ongoing efforts in the nanomaterial field to reduce process costs. Key aspects for future development include scaling up nanomaterial production, exploring in situ applications, and conducting further investigations into nanomaterial toxicity for safe implementation.

## Figures and Tables

**Figure 2a:**
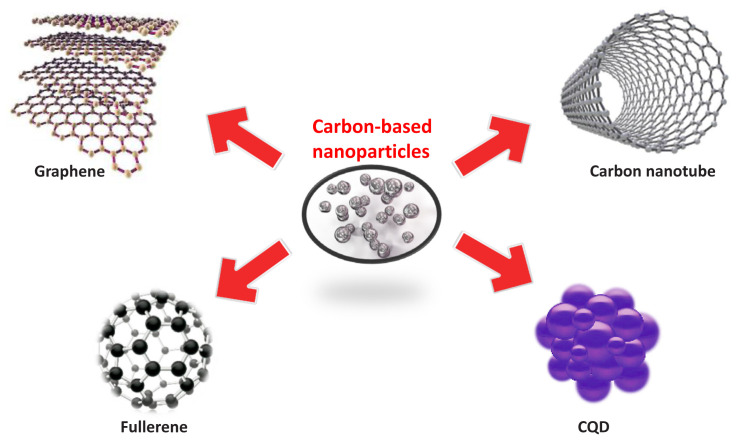
Carbon-based nanoparticles.

**Figure 2b:**
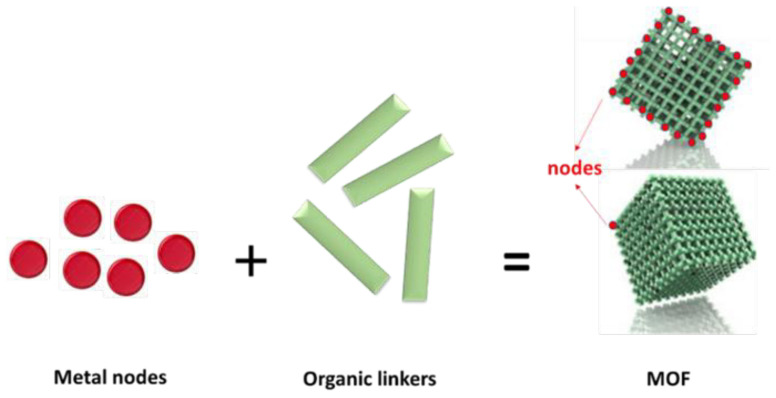
Schema of formation of MOFs.

**Figure 3a:**
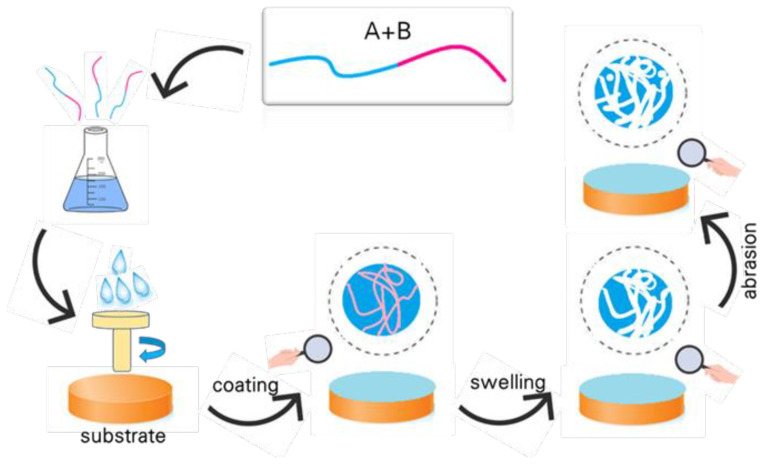
The mechanism of block copolymer formation.

**Figure 3b:**
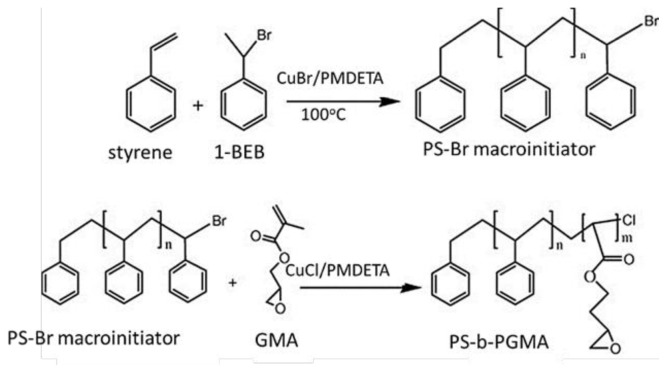
Schematic illustration of the synthesis of PS-*b*-PGMA diblock copolymer.

**Figure 3c:**
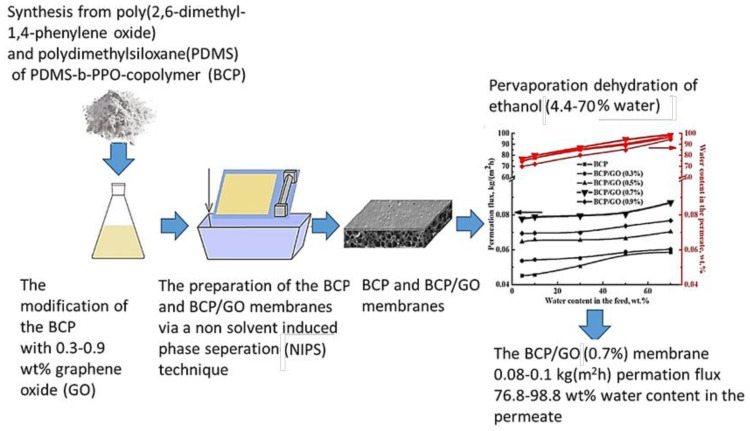
Graphical abstract of the study of Dmitrenko et al. [104].

**Table:** Comparison of properties of nanomaterial-based membranes [21–23].

Material	Pore size control	Mechanical strength	Ion selectivity	applicability	Low cost	Toxicity
**Polymeric membranes**	X	✓	✓	✓	✓	✓
**Block copolymers**	✓	X	✓	✓	X	X
**Metal-based membranes**	✓	X	✓	✓	✓	X
**Carbon-based membranes**	✓	✓	X	X	X	X
